# Correction: The integrated stress response induces R-loops and hinders replication fork progression

**DOI:** 10.1038/s41419-020-02817-y

**Published:** 2020-07-31

**Authors:** Josephine Ann Mun Yee Choo, Denise Schlösser, Valentina Manzini, Anna Magerhans, Matthias Dobbelstein

**Affiliations:** Institute of Molecular Oncology, Göttingen Center of Molecular Biosciences (GZMB), University Medical Center Göttingen, 37077 Göttingen, Germany

**Keywords:** DNA, Tumour-suppressor proteins

Correction to: *Cell Death and Disease*

10.1038/s41419-020-2727-2 published online 16 July 2020

In the original version of this Article, the figures were not in the correct sequence, and the references and legends did not match with the figures. This has now been corrected in the PDF and HTML versions of the Article.

